# Polymerized human hemoglobin with low and high oxygen affinity in trauma models

**DOI:** 10.1016/j.trsl.2023.05.006

**Published:** 2023-06-01

**Authors:** Cynthia R. Muller, Vasiliki Courelli, Cynthia Walser, Clayton T. Cuddington, Savannah R. Wolfe, Andre F. Palmer, Pedro Cabrales

**Affiliations:** 1 Department of Bioengineering, University of California San Diego, San Diego, CA; 2 Department of Chemical and Biomolecular Engineering, The Ohio State University, Columbus, OH

**Keywords:** Polymerized hemoglobin, Hemorrhagic shock, Oxygen carriers, Traumatic brain injury, Red blood cell substitute, Oxygen therapeutics

## Abstract

The present study aimed to compare the ability of tense (T) and relaxed (R) quaternary state polymerized human hemoglobin (PolyhHb) to restore hemodynamics after severe trauma in a rat model, and to assess their relative toxicity in a guinea pigs (GPs). To assess the efficacy of these PolyhHbs in restoring hemodynamics, Wistar rats were subjected to traumatic brain injury (TBI) followed by hemorrhagic shock (HS). Animals were separated into 3 groups based on the resuscitation solution: Whole blood, T-state or R-state PolyhHb, and followed for 2 hours after resuscitation. For toxicity evaluation, GPs were subjected to HS and the hypovolemic state was maintained for 50 minutes. Then, the GPs were divided randomly into 2 groups, and reperfused with T- or R-state PolyhHb. Rats resuscitated with blood and T-state PolyhHb had a higher recovery of MAP at 30 min after resuscitation when compared to R-state PolyhHb, demonstrating the greater ability of T-state PolyhHb to restore hemodynamics compared to R-state PolyhHb. Resuscitation with R-state PolyhHb in GPs increased markers of liver damage and inflammation, kidney injury and systemic inflammation compared to the T-state PolyhHb group. Finally, increased levels of cardiac damage markers, such as troponin were observed, indicating greater cardiac injury in GPs resuscitated with R-state PolyhHb. Therefore, our results showed that T-state PolyhHb exhibited superior efficacy in a model of TBI followed by HS in rats, and presented reduced vital organ toxicity in GPs, when compared to R-state PolyhHb.

## INTRODUCTION

As estimated by Jones et al. 10.6 million units of whole blood–derived and apheresis red blood cells (RBCs) were transfused in 2017, a 6.1% decline since 2015.^1^ Recently, the number of blood donations has been decreasing and sometimes fails to meet the demand, especially during shortages induced by seasonal epidemics as well as during pandemics as was observed recently during the COVID-19 pandemic.^1,2^ Blood transfusion is essential for treatment of trauma with penetrating injury, surgery, and for several hematological conditions. Therefore, an important goal of whole blood or RBC transfusion is the effective delivery of O_2_ to vital organs and restoration of blood volume (BV).^3^ In the case of loss of 30% to 40% of the BV, it is critical to restore BV immediately, to ensure that tissue perfusion and O_2_ supply is not compromised.^4^

In order to address the blood shortage issue, many efforts have been made in the last 2 decades to develop safe and efficacious RBC substitutes, such as hemoglobin (Hb)-based O_2_ carriers (HBOCs). It is well known that cell-free Hb cannot be used as an HBOC, since Hb can extravasate out of the circulation into the tissue space and elicit vasoconstriction, systemic hypertension, oxidative tissue damage, and cell death. However, chemically crosslinking Hb to form larger molecular diameter polymerized Hb (PolyHb) compared to Hb reduces Hb toxicity and enables PolyHb utilization as an HBOC.^5^ Hb changes its quaternary structure based on the presence or absence of O_2_ in the heme pocket of each of the 4 globin subunits. When Hb is bound to O_2_ at all 4 O_2_ binding sites, Hb is in the relaxed (R) quaternary state, while a deoxygenated Hb molecule is in the tense (T) quaternary state.

Hb can be either fully deoxygenated (pO_2_ = 0 mm Hg) and consists of 100% T-state Hb or fully oxygenated (pO_2_ = 760 mm Hg) and consists of 100% R-state before being chemically crosslinked to form PolyHb, resulting in either T-state PolyHb or R-state PolyHb, respectively.^6^ During Hb polymerization, the Hb molecule is locked in either of these 2 quaternary states thus allowing PolyHb to be synthesized in either the R or T quaternary state. The O_2_ affinity is quantified by the O_2_ tension (pO_2_) at which half of O_2_ binding sites on the Hb molecule are saturated with O_2_ (i.e., P_50_). T-state PolyHb has a high P_50_ (>30 mm Hg), while R-state PolyHb has a low P_50_ (<2 mm Hg). The high O_2_ affinity PolyHb conformation is in the R-state, while the low O_2_ affinity PolyHb is in the T-state. Therefore, T-state PolyHb will offload O_2_ over a wide range of O_2_ tensions, while R-state PolyHb will only offload O_2_ under anoxic conditions.^6^ T-state PolyHb favors precapillary O_2_ delivery to tissues, which can increase the O_2_ flux across the blood vessel wall, triggering vascular autoregulatory mechanisms to prevent O_2_ toxicity.^7^ However, oversupply of O_2_ by T-state PolyHb could also favor the formation of reactive O_2_ species (ROS). On the other hand, R-state PolyHb tends to tightly bind O_2_ and mostly releases O_2_ at the capillary level where tissue pO_2_ is low and the demand for O_2_ is high, thus preventing vascular hyperoxygenation and decreasing vasoconstriction and ROS production.

Our group has developed a high molecular weight (MW) T-state PolyHb synthesized with human Hb (PolyhHb) with increased MW compared to previous generations of commercial HBOCs, and has observed that the increased molecular size of PolyhHb decreases toxicity in Guinea pigs (GPs), which is a more clinically relevant model, since GPs like humans do not synthesize ascorbic acid and must take it in via dietary sources.^8^ Furthermore in a model of traumatic brain injury (TBI) followed by hemorrhagic shock (HS), T-state PolyhHb was shown to be efficacious at restoring hemodynamics, with similar results compared to fresh blood, and was superior compared to lactated Ringer’s solution.^9^ However, further studies are necessary in order to determine the most appropriate PolyhHb O_2_ affinity to be used for trauma resuscitation.

Our hypothesis is that selection of PolyhHb’s O_2_ affinity is dependent on the specific clinical application. For example, shock resuscitation demands rapid O_2_ delivery and T-state PolyhHb could be the appropriate molecule for resuscitation. However, in extreme scenarios with severe HS and or other associated traumas such as TBI, controlling O_2_ delivery could be a better option to avoid further oxidative stress damage, and also to ensure that the most hypoxic tissues receive proper amounts of O_2_. Therefore, the goal of the present study was to compare the ability of T-state and R-state PolyhHbs to restore hemodynamics after trauma in rats, and its’ toxicity 24 hours after resuscitation in GPs.

## MATERIAL AND METHODS

### Polymerized human hemoglobin

PolyhHb was synthesized in the low O_2_ affinity T-state at a 30:1 molar ratio of glutaraldehyde to human Hb (hHb), or in the high O_2_ affinity R-state at a 30:1 molar ratio of glutaraldehyde to hHb. The PolyhHb was then purified by passing it through a 0.2 *μ*m polyethersulfone hollow fiber filter cartridge (ID: S02-P20U-05-N, Repligen, Rancho Dominguez, CA) and subjected to 8–9 cycles of diafiltration on a polysulfone 500 kDa hollow fiber filter cartridge (ID: S02-P20U-05-N, Repligen, Rancho Dominguez, CA). This resulted in a PolyhHb solution containing only polymerized Hb molecules with MW greater than 500 kDa but less than 0.2 μm in size. The PolyhHb’s biophysical properties are shown in Table 1. PolyhHb was synthesized and characterized at The Ohio State University and shipped overnight frozen to University of California San Diego where it was stored at −80 C until use. The preparation and characterization of PolyhHb has been previously described in the literature.^10,11^

### Animal preparation for evaluating efficacy

Efficacy studies were performed in male Wistar rats weighing 350–400 g (Charles River Laboratories, Wilmington, MA). Animal handling and care followed the NIH Guide for Care and Use of Laboratory Animals, and all protocols were approved by the University of California San Diego Institutional Animal Care and Use Committee. All methods were carried out in accordance with ARRIVE guidelines (Animal Research: Reporting of In Vivo Experiments). Briefly, animals were anesthetized using isoflurane 5% in compressed room air for induction (Drägerwerk AG, Lübeck, Germany) and then maintained at 1.5%, except during surgical procedures when it was briefly increased to 2.5%. Animals were placed on a heating pad to preserve the core body temperature at 37°C and allowed to freely breathe from a nosecone delivering anesthesia. Animals were instrumented with a right femoral artery and vein catheter for hemodynamic assessment, and blood withdrawal and intravenous infusion, respectively. Animals were allowed to stabilize for 10 minutes and baseline measurements were collected.

### Traumatic brain injury

**Fig 1.**
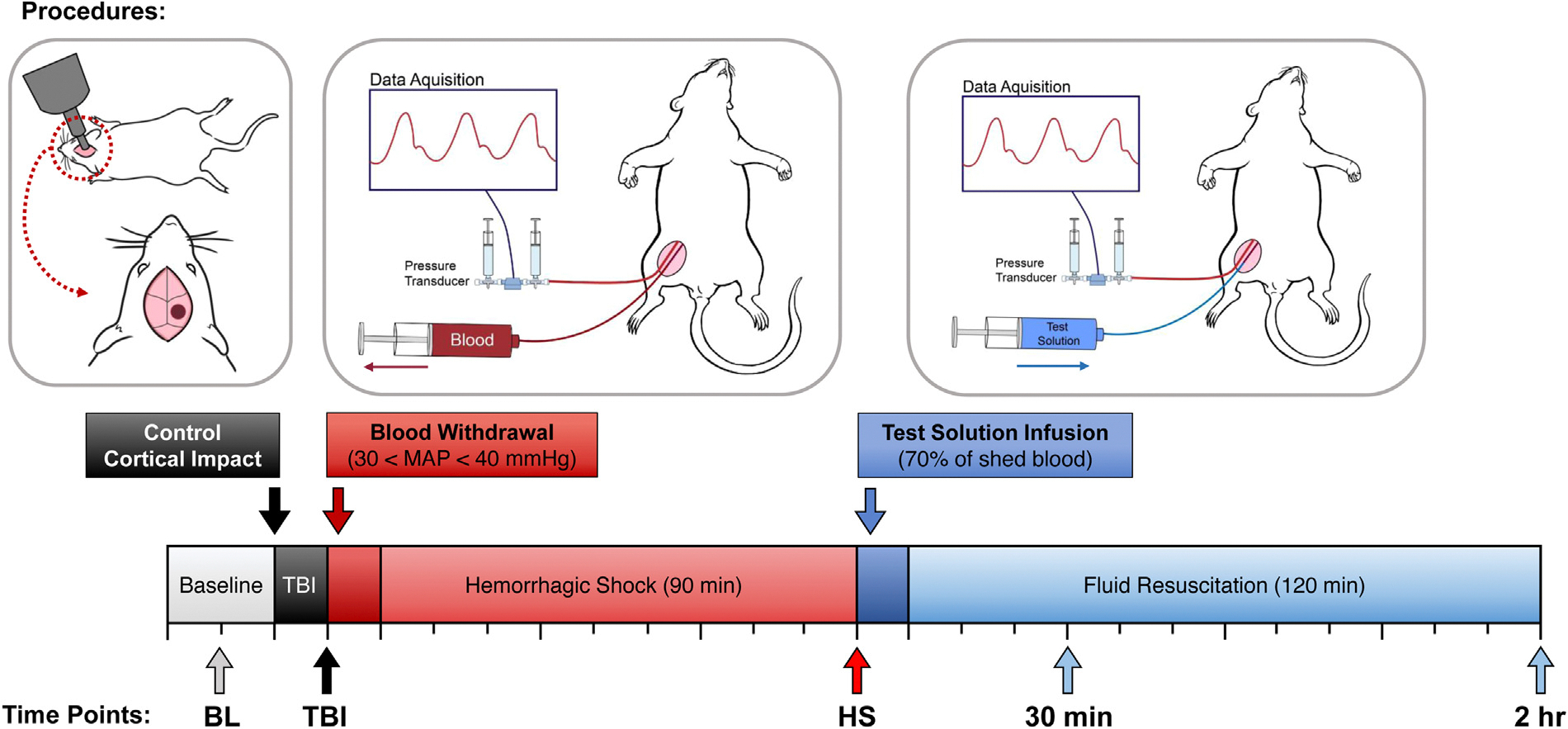
Time line for animal preparation for efficacy evaluation.

Animals were moved to a stereotaxic apparatus and placed in the ventral position, and the isoflurane was increased to 2.5% for 5 minutes before inducing TBI. To induce TBI, a 5 mm craniotomy was performed over the right cerebral cortex, and the dura was impacted with a 5.0 mm flat tipped impactor at a velocity of 5 m/s and dwell time of 200 ms via a pneumatically controlled cortical impactor (CCI; Leica Biosystems, Vista, CA). After CCI, the head was closed and the animals were placed back on the heating pad dorsally, and the isoflurane was decreased to 1.5%/vol for 10 minutes before starting hemorrhagic shock (HS) (Fig 1).^9^

### Hemorrhagic shock

All animals were given 10 minutes to stabilize after performing the TBI before performing HS. Animals were intravenously heparinized (100 IU/kg) to ensure patency of the catheters during the protocol. Hemorrhage was induced by removing blood from the femoral vein (0.5 mL/min) until the MAP reached 40 mmHg. The MAP was maintained between 35 and 40 mmHg for 90 minutes by withdrawing or returning small volumes of blood when the MAP was out of the indicated range for more than 2 minutes. After the prolonged severe hypovolemia, animals were randomly assigned to 1 of 3 groups (n = 6/group) based on the test intravenous resuscitation fluid: whole blood collected during the hemorrhage (total Hb concentration: approximately 11 g/dL), T-state PolyhHb (total Hb concentration: 10 g/dL), or R-state PolyhHb (total Hb concentration: 10 g/dL). All resuscitation solutions were administered intravenously at 2 mL/min, the total volume infused was 70% of the blood withdrawn during HS. Animals were monitored for 120 minutes from the beginning of the resuscitation period until euthanasia. Blood samples were taken at baseline (BL), 90 minutes into HS (HS), and 30 minutes and 2 hours after resuscitation. After samples were collected at the final time point, animals were euthanized via a single IV injection of sodium pentobarbital (300 mg/kg).^9^

### Systemic parameters

Arterial blood pressure and heart rate (HR) were recorded continuously from the femoral artery (MP150, Biopac, Santa Barbara, CA) at a 2 kHz sampling rate. Blood pressure recordings were used to calculate online mean arterial pressure (MAP), systolic blood pressure (SBP), and diastolic blood pressure (DBP) using AcqKnowledge software (Biopac). Hematocrit (HCT) was measured from centrifuged arterial blood samples taken in heparinized capillary tubes. Arterial and venous blood was collected in heparinized glass capillary tubes (65 μL) and immediately analyzed for O_2_ partial pressure (pO_2_), carbon dioxide partial pressure (pCO_2_), pH, Hb saturation, glucose, and lactate (ABL90; Radiometer America, Brea, CA).

### Inclusion criteria

Animals were suitable for experiments if: (i) MAP was greater than 85 mmHg at baseline, (ii) lactate less than 1.5 mmol/L at baseline, (iii) systemic Hb was greater than 12 g/dL at baseline, and (iv) animals survived the hypovolemic period.

### Metabolomics

Metabolomics analyses were performed on the rat brain impacted area after resuscitation. Metabolomics analyses were performed using an UPLC system (Ultimate 3000, Thermo, San Jose, CA) on a Kinetex XB-C18 column (Phenomenex, Torrance, CA) at 250 *μ*l/min. Briefly, 10 microliters of sample were injected into the UPLC system. Calibration was performed before each analysis against positive or negative ion mode calibration mixtures (Piercenet; Thermo, Rockford, IL). Metabolite assignments were performed using the software Maven (Princeton, NJ) and metabolite assignment against the KEGG database, and the library of standard compounds (Sigma-Aldrich, St. Louis, MO).

### Animal preparation for evaluation of tissue damage and toxicity

Animal handling and care followed the National Institutes of Health Guide for the Care and Use of Laboratory Animals, and the University of California San Diego Institutional Animal Care and Use Committee approved the experimental protocol. All methods were carried out in accordance with ARRIVE guidelines (Animal Research: Reporting of In Vivo Experiments). Guinea pigs (GPs) weighing between 300 g and 400 g were used. Animals were placed on a heating pad to maintain their core body temperature at 37°C for any procedures or experimental protocols that were performed under anesthesia. GPs were anesthetized with isoflurane (Drägerwerk AG, Lübeck, Germany) in compressed room air (flow rate 1.0 LPM) slowly, by increasing the isoflurane 0.4% every 3 minutes until a surgical depth of anesthesia was achieved, typically 3%. This ensured that the animals did not stop breathing due to airway irritation by isoflurane and prevented variations in HR. Animals were instrumented with catheters in the right carotid artery and left jugular vein, which were exteriorized dorsally.

### Hemorrhagic shock (HS)

HS was induced by 40% blood volume (BV) withdrawal from the carotid artery (BV estimated as 7.5% of body weight) and the hypovolemic state was maintained for 50 minutes. After being subjected to 50 minutes of HS, GPs were divided into 2 study groups, and 25% of their BV was reinfused with: T-state PolyhHb (total Hb concentration: 10 g/dL), or R-state PolyhHb (total Hb concentration: 10 g/dL).

### Hemodynamic and hematological measurements

The arterial cannula was connected to a pressure transducer and recording system (MP150, Biopac, Santa Barbara, CA), and blood signals were recorded, along with MAP, and HR. The HCT was measured from centrifuged arterial blood samples taken in heparinized capillary tubes. Arterial blood was collected in heparinized glass capillaries (50 *μ*L) and immediately analyzed for pO_2_, pCO_2_, pH, electrolytes, lactate, and Hb content (ABL90; Radiometer America, Brea, CA). All of these measurements were taken at baseline, 15 minutes after resuscitation, and animals were allowed to recover from anesthesia. Moreover, measurements were taken without anesthesia at 2 hours and 24 hours after resuscitation.

### Harvesting tissues

GPs were anesthetized and 10 mL of blood was collected from the indwelt arterial catheter and centrifuged to separate the plasma. GPs were euthanized with Fatal Plus (sodium pentobarbital, 300 mg/kg), and urine, kidneys, liver, spleen, and heart were harvested. Markers of inflammation, cardiac function, and organ injury were evaluated. These analyses were performed by the UC San Diego Histology Core via ELISA and flow cytometric analysis of tissue homogenates and plasma. The kits and methods used for these analyses are described in Supplemental Table 1.

### Statistical analysis

All values are expressed as the mean ± SE. Data was analyzed using 2-way analysis of variance (ANOVA) for repeated measurements of the parameters over time. When appropriate, post hoc analyses were performed with the Tukey multiple comparisons test. One-way ANOVA was used for the tissue measurement taken only at 24 hours. All statistics were calculated using GraphPad Prism 6 (GraphPad Software, Inc, San Diego, CA). Results were considered significant if *P* < 0.05.

## RESULTS

In general, all animals included in both models survived the experimental protocols and infusion of PolyhHb. There were no differences between groups at baseline, or after trauma.

## RAT MODEL OF TBI FOLLOWED BY HS RESUSCITATION

### Hemodynamics

**Fig 2.**
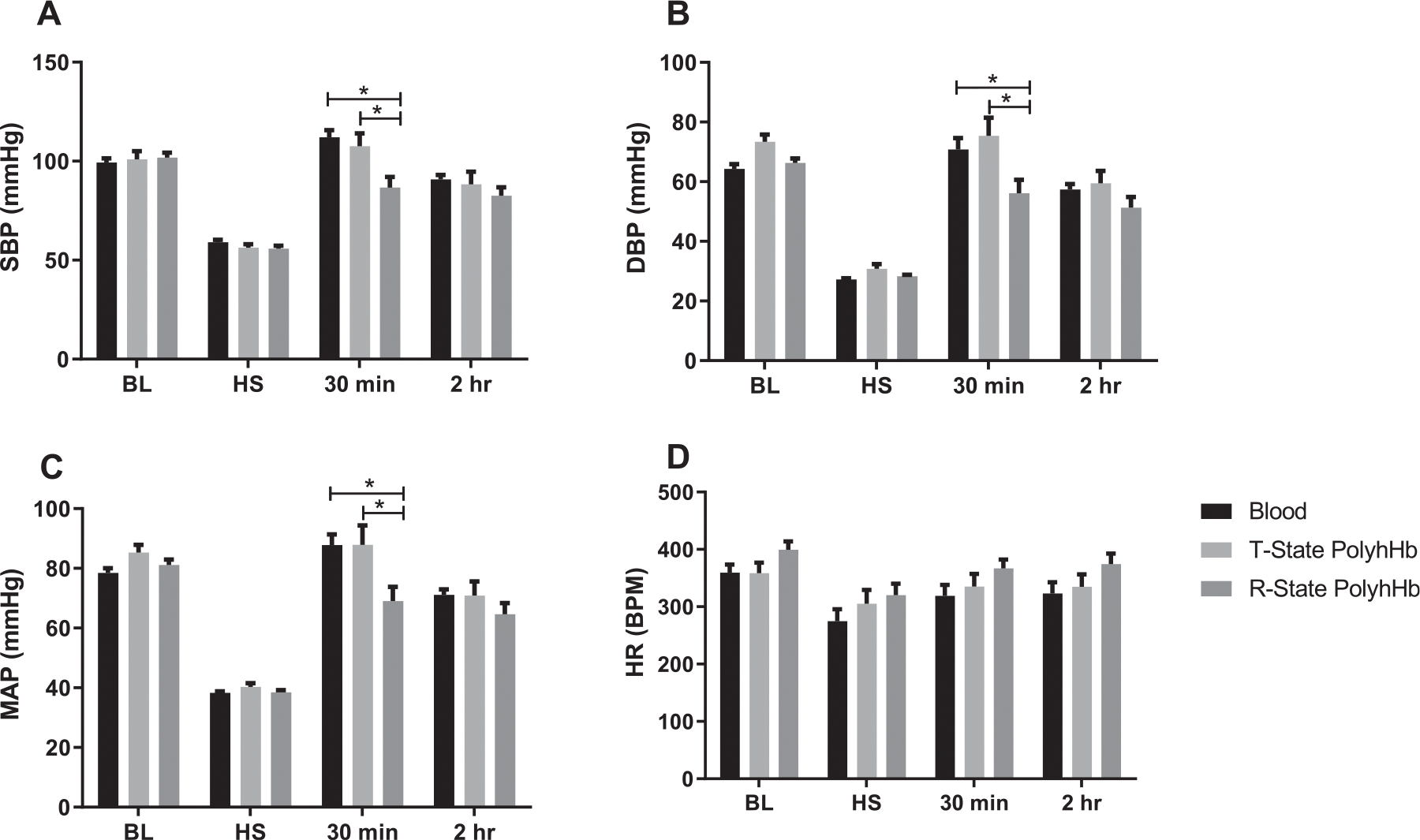
Blood pressure measurements of rats resuscitated with blood, PolyhHb T-state or R-state. **A**, SBP, systolic blood pressure. **B**, DBP, diastolic blood pressure. **C**, MAP, mean arterial pressure. **D**, HR, heart rate. *P* < 0.05. Blood (n = 6); T-state PolyhHb (n = 6); and R-state PolyhHb (n=6).

In this resuscitation model, we targeted infusion of 70% of the shed blood volume. It was observed that 30 minutes after fluid resuscitation, the R-state PolyhHb group presented lower SBP, DBP and MAP compared to the blood and T-state PolyhHb group. Remarkably, T-state PolyhHb presented the same blood pressure recovery compared to blood (Fig 2). No changes were observed among groups with respect to HR. There were no differences in blood pressure at 2 hours after resuscitation between groups.

### Blood gasses

In the TBI HS rat model, resuscitation with the T-state PolyhHb group increased arterial pO_2_ at 2 hours after resuscitation compared to resuscitation with blood, while both T- and R-state PolyhHbs presented lower pCO_2_ compared to blood. Finally, arterial O_2_ saturation was lower in the T-state PolyhHb group compared to both blood and R-state PolyhHb groups. However, 2 hours after resuscitation, there was no difference between the T-state PolyhHb and blood group. The R-state PolyhHb group presented higher arterial O_2_ saturation compared to both the blood and T-state PolyhHb groups. No changes were observed for pH, HCO_3,_ and base excess between groups at 2 hours after resuscitation (Table 2).

### Hematological parameters

All groups decreased the HCT after TBI with HS, but only the blood group recovered HCT after reperfusion. This was expected since both PolyhHb solutions are acellular solutions and do not contain RBCs. Total Hb decreased after TBI with HS, and all groups recovered Hb content to similar levels compared to baseline. No remarkable changes were observed with respect to blood glucose between groups within the 2 hours observation time (Table 2).

### Metabolomics

**Fig 3.**
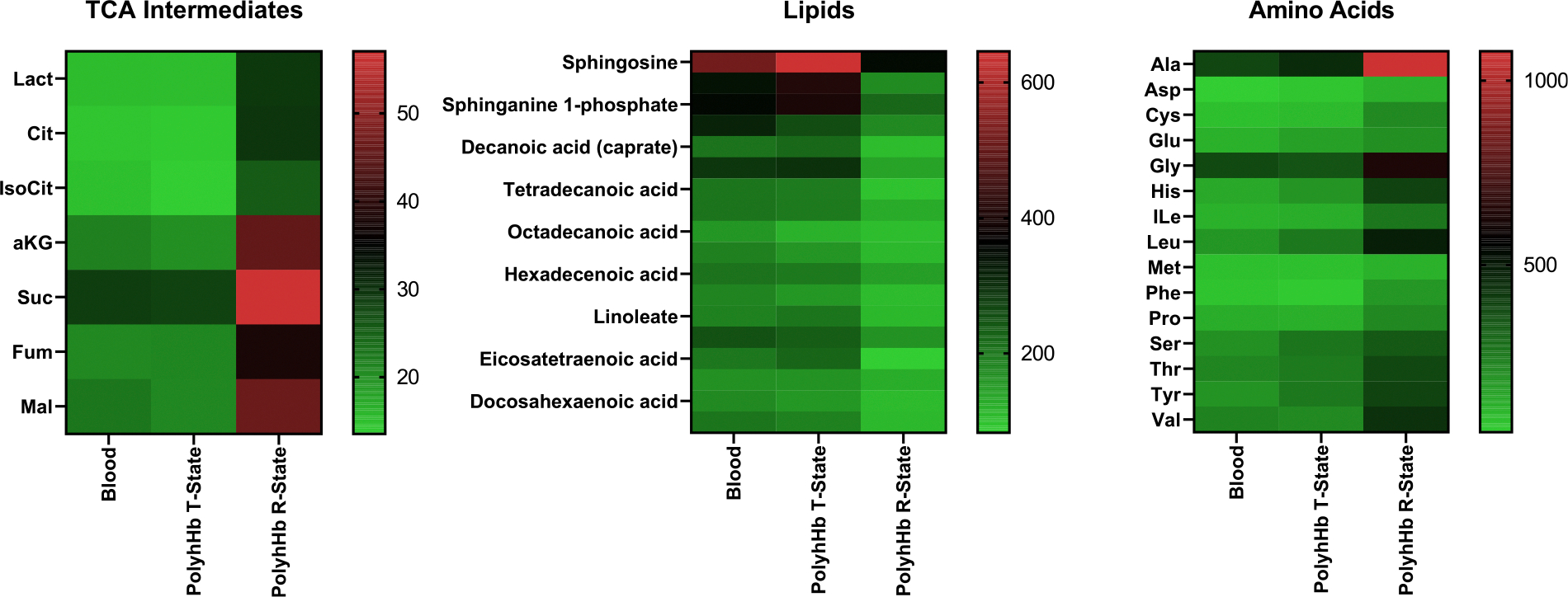
Metabolomics of TCA intermediates, lipids, and amino acids. Blood (n = 6); T-state PolyhHb (n = 6); and R-state PolyhHb (n = 6). Results are expressed by the median of each group.

Understanding the tricarboxylic acid (TCA) cycle, lipid and amino acid metabolites can lead to better outcomes in trauma involving TBI. In the present study, we evaluated brain metabolomics of the rats subjected to TBI with HS at 2 hours after resuscitation. In general, our results indicate that the metabolomics of the T-state PolyhHb group was similar to the blood group. On the other hand, R-state PolyhHb presented a 2 × - fold increase in accumulation of TCA intermediate metabolites and an increase in amino acids, and decrease of approximately 50% of lipid metabolites compared to blood and T-state PolyhHb (Fig 3).

## GP MODEL OF HS RESUSCITATION

Hemodynamic parameters were monitored during the entire protocol and vital tissue markers of function, inflammation, and injury were studied 24 hours after resuscitation.

### Hemodynamics

In this resuscitation model, both PolyhHb groups decreased blood pressure to similar levels during shock, and no differences were observed with respect to MAP restoration between groups after reperfusion. The HR was higher for the T-state PolyhHb group compared to the R-state PolyhHb group 2 hours into reperfusion. After 24 hours post resuscitation, and without anesthesia, there were no observed differences in MAP or HR between groups (Table 3).

### Vital tissue function, inflammation, and injury markers

**Fig 4.**
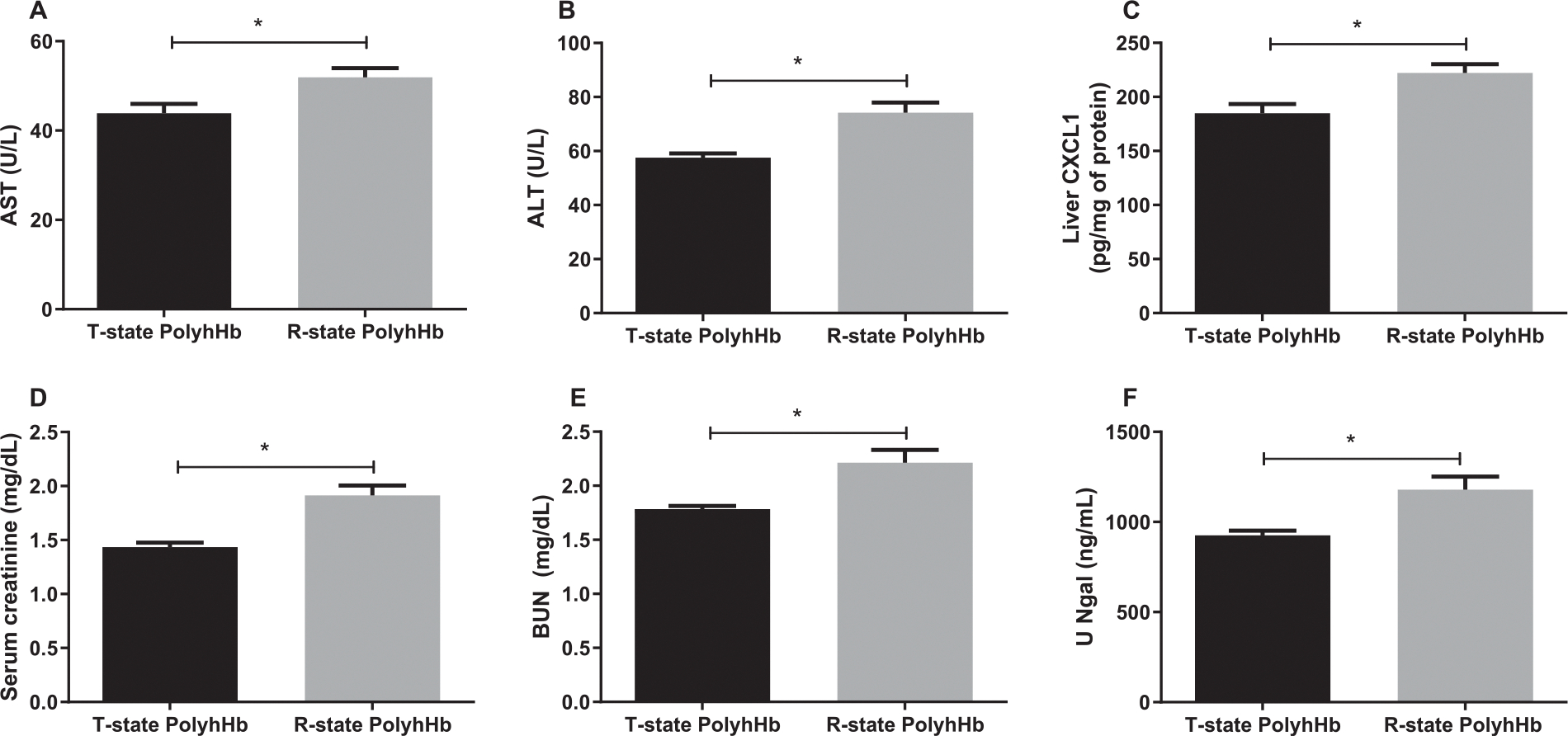
Liver and kidney injury 24 hours after resuscitation. **A**, AST, aspartate aminotransferase. **B**, ALT, alanine aminotransferase. **C**, (CXCL-1), liver chemokine ligand-1. **D**, serum creatinine. **E**, BUN, blood urea nitrogen; and **F**, urine neutrophil gelatinase-associated lipocalin (U-NGAL). *P < 0.05. T-state PolyhHb (n = 7); and R-state PolyhHb (n = 7).

In this resuscitation model, GPs resuscitated with R-state PolyhHb presented higher levels of AST (Fig 4A), ALT (Fig 4B), and liver CXCL1 (Fig 4C) compared to T-state PolyhHb. Moreover, GPs resuscitated with R-state PolyhHb exhibited increased markers of kidney injury as observed by increased serum creatinine (Fig 4D), BUN (Fig 4E) and urine Ngal (Fig 4F) compared to GPs resuscitated with T-state PolyhHb.

### Systemic inflammation and catecholamines

Markers of inflammation in GPs resuscitated with R-state PolyhHb were also higher compared to GPs resuscitated with T-state PolyhHb, as indicated by increases in systemic plasma IL-6, CXCL-1 and IL-10. Similarly, spleenic levels of CXCL1 were also increased in GPs resuscitated with R-state PolyhHb compared to in GPs resuscitated with T-state PolyhHb (Table 4). Finally, resuscitation with R-state PolyhHb also increased catecholamines (epinephrine and norepinephrine) compared to resuscitation with T-state PolyhHb in GPs (Table 4).

### Cardiac injury

**Fig 5.**
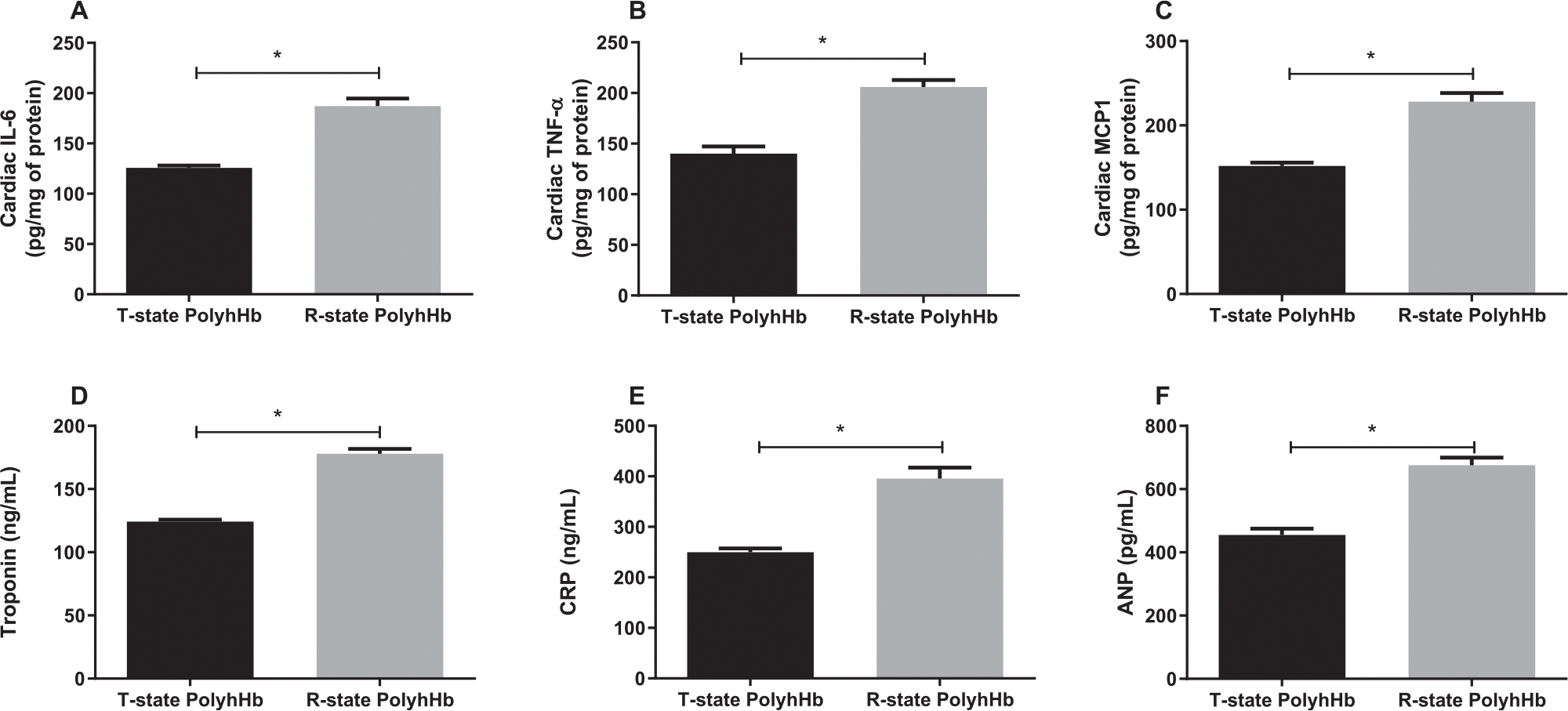
Cardiac inflammation and injury 24 hours after resuscitation. **A**, Cardiac interleukin-6 (IL-6). **B**, Cardiac tumor necrosis factor alpha (TNF-*α*). **C**, Cardiac monocyte chemoattractant protein-1 (MCP-1). **D**, Cardiac troponin. **E**, C-reactive protein; and **F**, ANP, atrial natriuretic peptide. *P < 0.05. T-state PolyhHb (n = 7); R-state PolyhHb (n = 7).

In this resuscitation model, GPs resuscitated with R-state PolyhHb showed a significant increase in cardiac markers of injury and inflammation compared to GPs resuscitated with T-state PolyhHb. Animals resuscitated with R-state PolyhHb showed increased cardiac IL-6 (Fig 5A), TNF-alpha (Fig 5B), MCP-1 (Fig 5C), troponin (Fig 5D), CRP (Fig 5E) and ANP (Fig 5F) compared to GPs resuscitated with T-state PolyhHb. These markers give us a fair, indirect observation of cardiac function and damage, showing that R-state PolyhHb is fairly detrimental towards cardiac tissues compared to T-state PolyhHb.

### Iron metabolism

**Fig 6.**
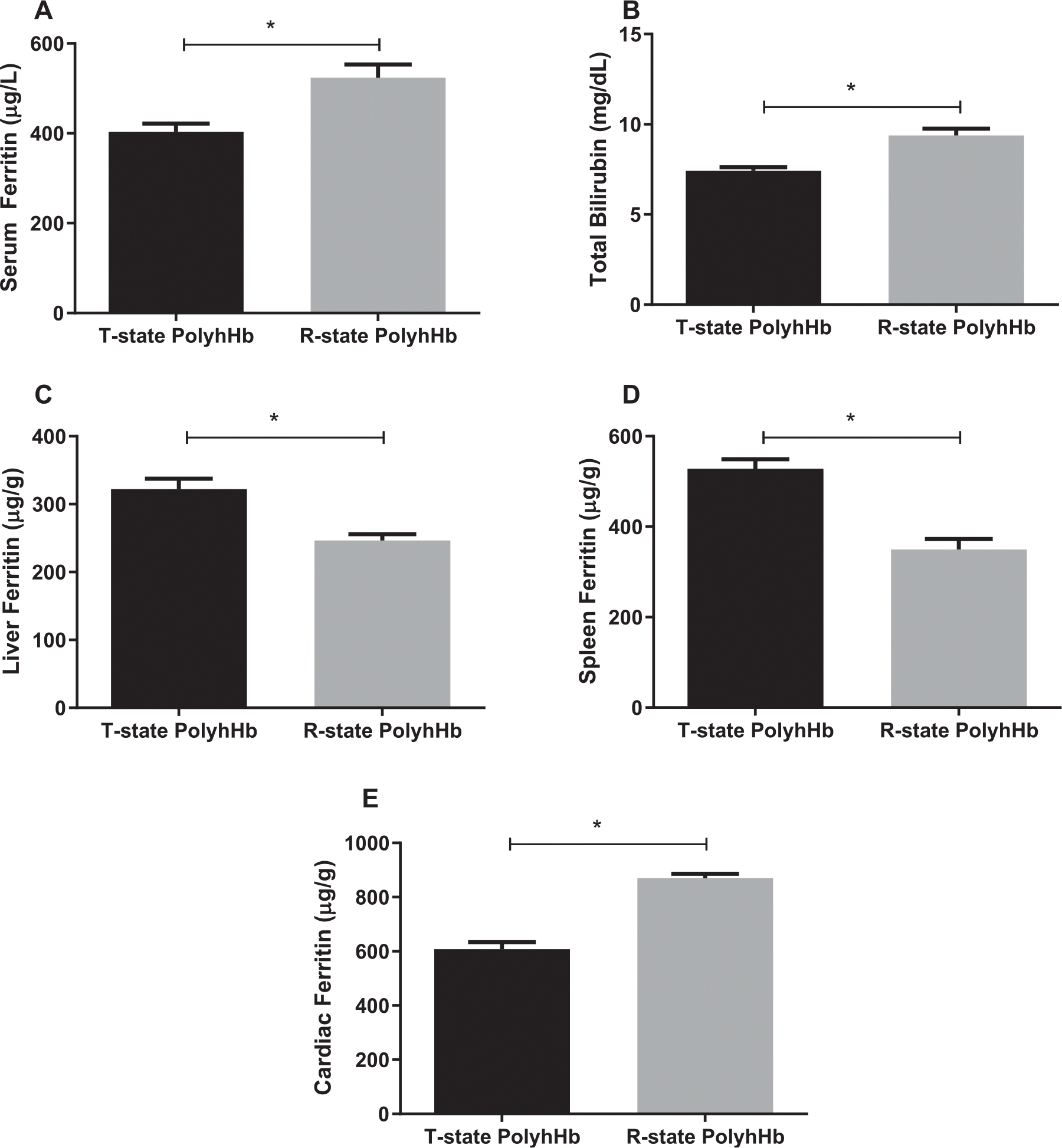
Iron metabolism measurement 24 hours after resuscitation. **A**, Serum ferritin. **B**, Total bilirubin. **C**, Liver ferritin. **D**, Splenic ferritin; and **E**, Cardiac ferritin. *P < 0.05. T-state PolyhHb (n = 7); and R-state PolyhHb (n = 7).

We evaluated ferritin and total bilirubin as markers of iron metabolism in GPs resuscitated with PolyhHbs. Our results indicate that serum ferritin (Fig 6A), and total bilirubin (Fig 6B) were increased in GPs resuscitated with R-state PolyhHb compared to GPs resuscitated with T-state PolyhHb. Conversely, liver (Fig 6C) and spleen ferritin (Fig 6D) levels were lower for R-state PolyhHb compared to T-state PolyhHb. Finally, cardiac ferritin (Fig 6E) was increased in GPs resuscitated with R-state PolyhHb compared to GPs resuscitated with T-state PolyhHb.

## DISCUSSION

The main findings in this study are that resuscitation with T-state PolyhHb restores systemic hemodynamics that is comparable with fresh blood, and its’ O_2_ transport properties appear to be beneficial for resuscitation in severe (rats subjected to TBI and HS) and mild (GPs subjected to HS) trauma models when compared to R-state PolyhHb. Furthermore, resuscitation with T-state PolyhHb appears to result in lower changes in, inflammation, injury and function of vital organs compared to R-state PolyhHb in the GP HS resuscitation model. It is important to note that in this study, we used 2 different animal models. The first was a rat model of TBI followed by HS. This model was used to address each solution’s ability to restore hemodynamics when compared to blood, since it is not clear what constitutes an adequate treatment for TBI followed by HS. Our primary objective with this protocol was to compare the efficacy of T-state and R-state PolyhHb in restoring hemodynamics, specifically in the context of a model involving TBI followed by HS. Furthermore, our aim was to compare the performance of these solutions with that of blood as a resuscitation solution relative to blood. The second model consisted of GPs subjected to only HS. This model was designed to evaluate the toxicity and inflammation induced by PolyhHb solutions. As previously reported in the literature GPs were used to assess PolyhHb toxicity, since GPs are a more clinically relevant animal model due their lack of ascorbic acid production and antioxidant status, which is similar to humans.^12,13^

### PolyhHb efficacy for restoring hemodynamics

In previous work, our group has studied several resuscitation fluids for their ability to restore blood volume and blood flow when blood is not available.^14–17^ Based on this past research, an effective resuscitation fluid needs to meet several physiological standards, such as the ability to provide adequate colloid osmotic pressure (COP), viscosity and more importantly high O_2_ carrying capacity.^14^ However, during reperfusion, O_2_ overload could also be harmful to tissues and vital organs.^5^ To address this concern, when PolyhHb was being developed as an HBOC it was hypothesized that controlling O_2_ affinity could reduce reperfusion injury by preventing O_2_ overload. In the present study, we evaluated PolyhHb synthesized in 2 different quaternary states: T-state PolyhHb (tense quaternary state with low O_2_ affinity) and R-state PolyhHb (relaxed quaternary state with high O_2_ affinity). We observed that in the model of TBI followed by HS, T-state PolyhHb restored blood pressure unlike R-state PolyhHb which did not have the same effect, as it was less efficient in restoring hemodynamics after reperfusion. R-state PolyhHb would deliver O_2_ mainly to organs with higher hypoxic status i.e. low pO_2_, and will tightly bind O_2_, mostly releasing O_2_ at the capillary level, where tissue pO_2_ is low and O_2_ is needed.^18^ This property could result in a lack of adequate O_2_ delivery, and explain R-state PolyhHb’s lower efficacy and higher toxicity. It is important to note that the positive hemodynamic effects appear to be transient, and both T-state and blood groups are similar to the R-state 2 hours after reperfusion. This observation can be attributed to the protocol we chose, which involved a single infusion at the beginning of resuscitation. However, further studies involving multiple or continuous infusions, as well as long-term measurements taken 24 to 72 hours later, are necessary to gain a comprehensive understanding.

Although singular injuries, including severe TBI and HS, are associated with higher mortality rates, the combination of multiple injuries further aggravates the outcome. This is caused by the complex posttraumatic immune response, which is a key driver of late post-injury complications and fatal outcome rates after trauma.^19,20^ Furthermore, when trauma involves TBI, therapeutic regiments are particularly challenging due to a loss of local and systemic autoregulatory mechanisms. In this scenario, fluid resuscitation that restores perfusion to ischemic tissues thus preventing hypoxia also has the potential to worsen TBI-related brain pathology, as fluid resuscitation following the loss of local and systemic autoregulatory mechanisms can lead to cerebral hemorrhage, edema, and diffuse swelling.

In our previous work, we demonstrated that T-state PolyhHb required a much lower volume of solution (less than 70% of the shed blood volume) for resuscitation to restore BP to a value of 65 mmHg or higher when compared to lactated Ringer’s for resuscitating rats subjected to TBI followed by HS. Based on those previous results, we decided in the present study, to use a fixed volume for the reperfusion regiment, which corresponded to 70% of the shed blood volume for all groups. We observed that T-state PolyhHb is more efficacious compared to R-state PolyhHb at restoring blood pressure with the same infusion volume.

In order to better understand possible brain metabolic damage associated with R- and T-state PolyhHb reperfusion, we evaluated TCA cycle metabolites, amino acids and lipid metabolites. Mitochondria are cellular organelles that generate ATP and metabolites for survival and growth, respectively. Recent studies showed that mitochondria release TCA cycle metabolites to control cell fate and function. TCA cycle metabolites are byproducts of cellular metabolism and are important for biosynthesis of macromolecules such as nucleotides, lipids, and proteins. TCA cycle metabolites are also involved in controlling chromatin modifications, DNA methylation, and post-translational modifications of proteins to alter their function,^21^ in this scenario the accumulation of TCA cycle metabolites in the R-state PolyhHb group could indicate mitochondrial dysfunction, and could be causing further brain damage in animals resuscitated with R-state PolyhHb, however we cannot confirm that this TCA cycle alteration is causing mitochondrial dysfunction since we did not measure any marker of it, and future studies are necessary to confirm this hypothesis.

Amino acids metabolites also increased in the R-state PolyhHb group compared to the blood group, once more suggesting that animals resuscitated with R-state PolyhHb display brain metabolism dysfunction. A postmortem brain of an Alzheimer’s disease study showed a similar amino acid metabolomic profile to what was observed in the R-state PolyhHb group,^22^ once more reinforcing the fact that using R-state PolyhHb to resuscitate an animal in a model of TBI followed by HS could be causing further brain damage.

The crucial role of lipids in brain tissue pathophysiology is demonstrated by the deregulated lipid metabolism present in many neurological disorders, including bipolar disorders such as schizophrenia, and neurodegenerative diseases such as Alzheimer’s, and Parkinson’s, and also in the TBI.^23^ In fact, Djebali et al. demonstrated that neurosteroids such as progesterone and allopregnanolone reduced cell death, astrogliosis, and functional deficits in rats after TBI.^24^ In our model, the lipid metabolome was mostly reduced in animals resuscitated with R-state PolyhHb when compared to the blood group, suggesting a dysregulated lipid metabolism in this group. Taken together our metabolomic results suggest that resuscitation with R-state PolyhHb could be causing further brain metabolic injury, compared to T-state PolyhHb which behaves very similarly to blood, the gold standard resuscitation treatment. However, further analysis is necessary in order to determine if resuscitating animals with T-state PolyhHb is not causing further neurological damage.

### PolyhHb safety profile and toxicity

To assess the safety profile of R-state compared to T-state, we employed the GP model of HS. In previous studies, we demonstrated that T-state PolyhHb was equally effective as fresh blood in resuscitating GPs from HS, while exhibiting reduced toxicity compared to stored blood.^25^ Hence, in the current study, our primary objective using the GP model was to compare the toxicity of R-state PolyhHb with T-state PolyhHb. The pathogenesis of organ injury after trauma is a multifactorial process. The concomitant dysfunction of the endothelium, coagulation system, and intense proinflammatory response promotes secondary damage to tissues causing multiorgan dysfunction, and represents the leading cause of “late” death in trauma.^26^ Therefore, a certain level of inflammation is expected after HS and resuscitation, ideally the resuscitation solution should be able to decrease, or at least prevent further inflammatory responses to avoid further organ injury. In the present study, we investigated markers of inflammation and organ damage, and observed that R-state PolyhHb promotes higher inflammation, and vital organ injury, observed by kidney, liver and cardiac organ injury markers compared to T-state PolyhHb. Interestingly, in our previous study with bovine derived HBOCs comparing R-state PolyHb versus T-state PolyHb, these important differences in inflammation and organ toxicity were not observed; however, that study was performed in a model of 20% exchange transfusion.^27^ Therefore, we can infer that the solution itself does not cause inflammation or organ toxicity. However, the solution is not adequate at avoiding inflammation and organ injury in a severe trauma model. Our hypothesis is that the higher O_2_ affinity of R-state PolyhHb prevents the full recovery of O_2_ supply to vital organs preserving the shock hypoxic conditions, thus causing more damage and inflammation. Another, possibility to the limited efficacy of R-state PolyhHb is that the increase in O_2_ affinity increases the intravascular content of O_2_ and triggers an O_2_ autoregulatory mechanism that reduces blood flow and prevents adequate reperfusion. In future studies, we intend to explore the mechanisms responsible for the limited efficacy of R-state PolyhHb.

In the early stages of hemorrhage, decreased blood pressure and blood volume activate peripheral and central baro- and volume-receptors and trigger compensatory sympathetic and endocrine mechanisms. Sympathetic activation induces tachycardia and vasoconstriction of the ischemia-tolerant structures, increasing myocardial O_2_ consumption. Cerebral and cardiac autoregulation provides sufficient blood supply, however further blood loss exhausts compensatory mechanisms leading to hypoxia. The brain’s response to hypoxia further activates the sympathetic system.^28^ In our model of HS in GPs resuscitated with T-state PolyhHb or R-state PolyhHb, we measured catecholamines as markers of sympathetic activation, and observed that animals resuscitated with R-state PolyhHb presented higher levels of epinephrine and norepinephrine suggesting higher sympathetic activation compared to T-state PolyhHb. Once more these results could be correlated to higher levels of hypoxia. In this case, hypoxia could be happening for 2 reasons, because blood pressure remained low therefore decreasing blood flow to vital organs, or because of the solution’s high O_2_ affinity which impairs blood flow and O_2_ delivery.

Another common problem associated with blood and blood product transfusion, is iron toxicity.^29^ Iron plays a vital role in several cellular processes such as DNA synthesis, nucleic acid repair, cellular respiration in mitochondria, cell growth and cell death and contributes to host defense and cell signaling processes.^30–32^ Moreover, iron incorporated into heme is the main component of Hb and is thus crucial for O_2_ transport and supply by RBCs. Even though iron is essential for life, iron also has the potential to be toxic in the presence of hydrogen peroxide (H_2_O_2_). The liver, especially hepatocytes, play a major role in iron metabolism, because hepatocytes are able to synthesize a large amount of the iron storage protein ferritin. Due to this fact, hepatocytes act as major storage location for absorbed iron. In this context, the liver is the main organ responsible for iron clearance, when it is in excess.^33^ It is surprising to note that GPs resuscitated with R-state PolyhHb possess lower levels of liver ferritin compared to T-state PolyhHb resuscitated animals. On the other hand, cardiac ferritin was higher in the R-state PolyhHb group compared to the T-state PolyhHb group. This result suggests that when the liver could not clear the excess iron after transfusion of R-state PolyhHb, iron started to accumulate in other organs such as the heart, increasing the ferritin levels in that tissue. However possibly even after the increase in cardiac ferritin, additional iron accumulates in the tissue and could be causing further damage, which could explain the increased levels of cardiac injury markers observed in the R-state PolyhHb group compared to the T-state PolyhHb group.

Although R-state PolyhHb does not seem to be an adequate choice for trauma resuscitation, it could be a better choice for the treatment of other pathologies. For example, the importance of high O_2_ affinity Hb has been discussed during hypoxia and high altitude acclimatation in humans.^34^ In a healthy subject, the systemic decrease in Hb O_2_ affinity would compromise O_2_ loading in the lungs, particularly when O_2_ availability is limited during hypoxia. At higher altitudes, a decrease in Hb O_2_ affinity would be even more disadvantageous and further compromise O_2_ loading which would likely impede peripheral O_2_ delivery. Conversely, an increase in Hb O_2_ affinity during hypoxia promotes O_2_ loading within the lungs and mitigates reductions in arterial O_2_ saturation.^34^ Future studies should be performed in order to determine the role of this high O_2_ affinity PolyhHb to overcome issues in diverse pathologies.

## CONCLUSION

Taken together, these results suggest that T-state PolyhHb has better efficacy at restoring hemodynamics in a severe model of TBI followed by HS compared to R-state PolyhHb. Furthermore, it performs similarly to blood at restoring hemodynamics. Our study also showed that R-state PolyhHb exhibits higher vital organ toxicity. In this scenario, T-state PolyhHb is apparently a better choice when treating trauma. However, we hypothesize that R-state PolyhHb still can present higher performance when used to treat other pathologies, and more studies are necessary to confirm if R-state PolyhHb could be an alternative for treatment in other clinical scenarios. We believe that most of the unexpected effects observed in animals resuscitated with R-state PolyhHb is associated with hypoxia, but further investigation is necessary to better understand this pathophysiological response. Finally, R-state PolyhHb should still be studied in other models where targeted organ O_2_ delivery is important.

## Supplementary Material

supplemental

Supplementary material associated with this article can be found, in the online version, at doi:10.1016/j.trsl.2023.05.006.

## Figures and Tables

**Table 1:** Biophysical properties of hHb, T-state PolyhHb, and R-state PolyhHb

Solution	Glutaraldehyde to hHb molar ratio	Protein concentration (mg/mL)	MetHb level (%)	Average molecular weight (kDa)	P_50_ (mm Hg)	k_off,O2_ (s^−1^)	k_Hb-Hp_(s^−1^μM^−1^)

Unmodified hHb (n = 7)	0:1	235 ± 11	1.1 ± 0.4	64.5 ± 0.3	13.2 ± 0.6	36.4 ± 1.1	0.341 ± 0.006
30:1 T-state PolyhHb (n = 20)	30:1	106 ± 6	5.8 ± 1.6	1290 ± 100	41.3 ±3.7	46.3 ± 2.0	0.0124 ± 0.002
30:1 R-state PolyhHb (n = 2)	30:1	101 ± 1	3.7 ± 0.4	1320 ± 50	1.2 ± 0.4	13.2 ± 0.2	0.0062 ± 0.003

**Table 2:** Rat TBI+HS hematological parameters

		Blood	T-State PolyhHb	R-State PolyhHb

pH	BL	7.366 ± 0.004	7.387 ± 0.0128	7.368 ± 0.012
	HS	7.342 ± 0.011	7.364 ± 0.0176	7.364 ± 0.015
	30 min	7.374 ± 0.013	7.389 ± 0.0126	7.390 ± 0.017
	2 hr	7.390 ± 0.014	7.410 ± 0.0138	7.387 ± 0.026
pCO_2_ (mmHg)	BL	46.4 ± 2.3	45.3 ± 2.2	43.2 ± 1.3
	HS	33.8 ± 1.6	30.2 ± 1.5	35.4 ± 1.9
	30 min	41.2 ± 1.0	36.7 ± 2.2	36.5 ± 0.8
	2 hr	41.4 ± 2.5	33.8 ± 2.4^†^	31.3 ± 0.9^†^
pO_2_ (mmHg)	BL	80.7 ± 3.5	90.4 ± 7.2	78.1 ± 1.2
	HS	108.0 ± 2.8	119.4 ± 2.8	108.2 ± 6.2
	30 min	87.4 ± 3.5	98.6 ± 6.1	95.6 ± 3.8
	2 hr	83.5 ± 6.2	102.0 ± 4.9^†^	98.4 ± 3.2
HCO_3_ (mmol/L)	BL	24.3 ± 0.8	25.6 ± 0.5	24.1 ± 24.1
	HS	19.371 ± 1.0	18.5 ± 0.8	21.1 ± 21.1
	30 min	23.857 ± 0.6	22.4 ± 1.3	23.1 ± 23.1
	2 hr	24.086 ± 0.8	22.5 ± 1.4	21.2 ± 21.2
BE (mmol/L)	BL	0.43 ± 1.26	2.03 ± 0.58	0.13 ± 0.8
	HS	−6.86 ± 1.37	−8.36 ± 1.16	−4.41 ± 1.5
	30 min	−0.37 ± 0.84	−2.70 ± 1.76	−1.83 ± 1.5
	2 hr	−0.17 ± 1.08	−2.74 ± 1.97	−4.67 ± 2.0
SO_2_ (%)	BL	89.5 ± 1.1	89.9 ± 1.2	89.4 ± 0.5
	HS	95.7 ± 0.3	96.5 ± 0.3	95.2 ± 0.7
	30 min	91.7 ± 0.9	86.9 ± 1.2^†^	93.9 ± 1.2*
	2 hr	88.9 ± 3.6	87.5 ± 0.4	95.6 ± 1.1^†^,*
Lactate (mmol/L)	BL	0.8 ± 0.1	1.0 ± 0.1	0.9 ± 0.1
	HS	4.7 ± 0.5	6.3 ± 0.2	4.1 ± 0.6*
	30 min	1.4 ± 0.2	4.3 ± 0.6^†^	4.0 ± 0.8^†^
	2 hr	1.0 ± 0.1	3.3 ± 0.8^†^	4.6 ± 0.8^†^
HCT (%)	BL	42.3 ± 1.6	43.0 ± 1.9	41.0 ± 0.3
	HS	33.6 ± 1.1	31.7 ± 0.8	31.2 ± 0.9
	30 min	40.0 ± 1.2	26.7 ± 0.8^†^	28.6 ± 1.3^†^
	2 hr	41.2 ± 0.6	25.0 ± 0.9^†^	28.0 ± 1.3^†^
tHb (g/dL)	BL	14.0 ± 0.2	14.3 ± 0.2	13.2 ± 0.2
	HS	10.0 ± 0.2	10.3 ± 0.2	9.9 ± 0.2
	30 min	12.7 ± 0.3	12.4 ± 0.2	12.1 ± 0.1
	2 hr	11.1 ± 1.5	11.6 ± 0.3	11.8 ± 0.2
Glucose (mg/dL)	BL	228.0 ± 11.5	222.6 ± 26.0	216.9 ± 13.8
	HS	344.7 ± 47.4	360.3 ± 34.5	315.0 ± 19.2
	30 min	268.7 ± 26.8	275.3 ± 35.2	296.4 ± 36.6
	2 hr	219.9 ± 23.6	180.1 ± 29.5	181.1 ± 46.3

Data presented as mean ± SE.

* *P* < 0.05 compared to T-state. Blood (n = 6); T-state (n = 6); R-state (n = 6).

† *P* < 0.05 compared to blood.

**Table 3:** Guinea pig hemodynamics and hematological parameters

		T-state PolyhHb	R-state PolyhHb

MAP (mmHg)	BL	54.7±1.3	57.2±2.1
	Shock 50	36.3±1.3	34.3±2.3
	Rep 2hr	64.8±4.8	63.1±1.8
	Rep 24hr	76.2±2.6	78.4±1.7
HR (BPM)	BL	283.8±4.8	252.9±6.3
	Shock 50	281.0±3.9	256.0±5.2
	Rep 2hr	351.0±17.9	290.1±8.2*
	Rep 24hr	365.5±14.8	351.0±12.7
pCO_2_ (mmHg)	BL	50.1±1.7	57.6±4.9
	Shock 50	42.6±1.6	50.1±3.3
	Rep 2hr	29.6±1.9	38.9±0.9
	Rep 24hr	39.2±1.2	39.9±1.8
pO_2_ (mmHg)	BL	89.1±9.1	100.0±8.0
	Shock 50	103.1±4.8	103.1±2.4
	Rep 2hr	94.9±2.4	106.9±4.3
	Rep 24hr	74.5±3.4	67.8±3.1
SO_2_ (%)	BL	100.3±4.3	103.1±3.8
	Shock 50	107.9±1.1	108.0±0.7
	Rep 2hr	99.6±1.6	106.7±0.2
	Rep 24hr	101.1±1.9	104.4±1.4
Glucose (mg/dL)	BL	260.8±31.4	271.1±15.3
	Shock 50	276.3±46.6	237.3±30.7
	Rep 2hr	193.2±53.5	115.6±7.0
	Rep 24hr	148.6±6.1	148.8±9.4
Lactate (mmol/L)	BL	1.8±0.5	3.2±0.7
	Shock 50	3.1±0.5	3.5±0.5
	Rep 2hr	2.8±0.8	3.0±0.2
	Rep 24hr	1.1±0.2	1.1±0.1
HCT (%)	BL	41.2±0.5	41.4±0.6
	Shock 50	29.2±0.3	30.4±0.9
	Rep 2hr	27.2±0.9	25.6±0.5
	Rep 24hr	21.5±0.3	21.7±0.7
tHb (g/dL)	BL	12.3±0.4	12.5±0.4
	Shock 50	9.2±0.3	9.0±0.2
	Rep 2hr	10.9±0.4	10.6±0.1
	Rep 24hr	7.5±0.3	7.8±0.2

Data presented as mean ± SE.

* *P* < 0.05 compared to T-state. T-state (n = 7); R-state (n = 7).

**Table 4:** Guinea pig splenic and systemic inflammation and catecholamines

	T-state PolyhHb	R-state PolyhHb

Spleen CXCL1 (pg/mg of protein)	456.6±13.7	582.3±24.5*
Serum IL-6 (pg/mL)	325.7±18.7	408.4±16.0*
Serum CXCL-1 (pg/mL)	244.4±6.8	298.4±14.36*
Serum IL-10 (pg/mL)	296.8±9.7	388.3±15.0*
Norepinephrine (pg/mL)	828.5±56.5	1139.0±56.5*
Epinephrine (pg/mL)	364.9±5.7	498.1±24.1*

Data presented as mean ± SE.

* *P* < 0.05 compared to T-state. T-state (n = 7); R-state (n = 7).

## At A Glance Commentary

Muller C R, et al.

## Background

Herein, we report the effects of polymerized human hemoglobin (PolyhHb) quaternary state on resuscitation efficacy from traumatic brain injury (TBI) followed by hemorrhagic shock (HS).

## Translational Significance

The tense quaternary state PolyhHb evaluated in the present study has shown promising results and has high translational application since tense quaternary state PolyhHb could be used for trauma resuscitation when blood is unavailable.
