# Supplementary material for: Polymerized human hemoglobin with low and high oxygen affinity in trauma models
Source: Transl Res. Author manuscript; Available in PMC 2026 Feb 16. (PMC12908438; doi:10.1016/j.trsl.2023.05.006)
Supplement: supplemental [file NIHMS2138958-supplement-supplemental.docx]

| **Supplementary Table 1**: ELISA kits used in the analysis**.** | | | |
| --- | --- | --- | --- |
| **Method** | **Kit/Assay** | **Analyte** | **Vendor** |
| ELISA | KA1625 | AST | Abnova Corp, Taiwan |
| ELISA | KA4189 | ALT | Abnova Corp, Taiwan |
| ELISA | BMS625 | IL-6 | Thermo Fisher, Waltham, MA |
| ELISA | BMS629 | IL-10 | Thermo Fisher, Waltham, MA |
| ELISA | KB02-H2 | Creatinine | Arbor Assays Inc, Ann Arbor, MI |
| ELISA | K024-H5 | BUN | Arbor Assays Inc, Ann Arbor, MI |
| ELISA | ERCXCL1 | CXCL1 | Thermo Fisher, Waltham, MA |
| ELISA | MCA-155 | Ferritin | Serotec, Oxford, UK |
| ELISA | BA-E-6600 | Catecholamines | ImmunoSmol, France |
| ELISA | ERLCN2 | Urine NGAL | Thermo Fisher, Waltham, MA |
| ELISA | ab235627 | Bilirubin | Abcam, Cambridge, UK |
| Luminex technology /Miliplex Immunoassay | RECYTNMAG-65K | MCP-1 | Millipore Corporation, Massachusetts, USA |
| Luminex technology /Miliplex Immunoassay | RECYTNMAG-65K | TNF-α | Millipore Corporation, Massachusetts, USA |
| ELISA | ab246529 | Cardiac Troponin | Abcam, Cambridge, UK |
| ELISA | Ab256398 | CRP | Abcam, Cambridge, UK |
| ELISA | Ab108797 | ANP | Abcam, Cambridge, UK |
